# Draft Genome Sequence of a Lanthanide-Responsive Bacterium, *Bradyrhizobium* sp. Strain Ce-3

**DOI:** 10.1128/mra.00180-22

**Published:** 2022-06-23

**Authors:** Kohei Nakamura, Viagian Pastawan, Soya Suganuma, Kosuke Mizuno, Masaya Shimada, Takashi Hayakawa, Nanung Agus Fitriyanto, Tomoyuki Nakagawa

**Affiliations:** a Faculty of Applied Biological Sciences, Gifu University, Gifu, Japan; b The Graduate School of Natural Sciences and Technologies, Gifu University, Gifu, Japan; c Faculty of Animal Science, Universitas Gadjah Mada, Yogyakarta, Indonesia; d Department of Applied Life Studies, College of Nagoya Women’s University, Aichi, Japan; University of Southern California

## Abstract

We present the draft genome sequence of *Bradyrhizobium* sp. strain Ce-3, which produced exopolysaccharide (EPS) and oxidized methanol in the presence of Ce^3+^. The genome for strain Ce-3 was estimated at 7,608,996 bp and showed that the strain is closely related to Bradyrhizobium erythrophlei MT12 and *Bradyrhizobium* sp. strain C9.

## ANNOUNCEMENT

*Bradyrhizobium* sp. strain Ce-3 (= MAFF 211645) shows unique phenotypes depending on the presence of Ce^3+^, such as production of exopolysaccharide (EPS) (1) and induction of a lanthanide (Ln)-dependent methanol dehydrogenase (MDH), XoxF (2–4). In this study, we report the draft genome sequence of *Bradyrhizobium* sp. strain Ce-3.

Genomic DNA from strain Ce-3, which had been grown on hypo medium containing succinate as a carbon source by shaking culture at 28°C (3), was isolated using the NucleoSpin Soil kit (Macherey-Nagel GmbH & Co. KG, Düren, Germany). A library was constructed following the manufacturer’s protocol, with reagents supplied in the Nextera DNA Flex library preparation kit (Illumina, San Diego, CA). The library was sequenced with a MiSeq system (Illumina), producing 4,155,922 paired-end reads of 300 nucleotides. Default parameters were used for all software unless otherwise specified. Raw reads were processed with fastp v0.21.0 to remove low-quality reads and bases from reads (5). Quality-filtered reads (584,390,237 bp in total) giving coverage of 61× (calculated on the basis of an estimated genome size of 9.5 Mbp for Bradyrhizobium elkanii USDA 61) were assembled using SPAdes v3.15.2 (6). After removal of contigs shorter than 200 bp in length, the statistical summary of the final assembly was done by QUAST v5.0.2 (7). The assembly contained 98 scaffolds with a total length of 7,608,996 bp, with the largest contig of 1,394,038 bp, an *N*_50_ value of 671,729 bp, and a GC content of 65.02%. Annotation of the genome performed with DFAST (8) revealed 7,049 genes, which were identified as 6,997 protein coding sequences, 3 rRNAs, 48 tRNAs, and 1 CRISPR.

The comparison of 16S rRNA gene sequences was performed by EzBioCloud (9). The 16S rRNA gene of the strain Ce-3 genome revealed close phylogenetic relationships with B. elkanii USDA 76 (GenBank accession number KB900701), Bradyrhizobium pachyrhizi PAC 48 (GenBank accession number LFIQ01000091), Bradyrhizobium tropiciagri SEMIA 6148 (GenBank accession number LFLZ01000084), Bradyrhizobium brasilense UFLA 03-321 (GenBank accession number MPVQ01000039), and Bradyrhizobium ripae WR4 (GenBank accession number MF593081), the 16S rRNA genes of which are identical to that of Ce-3. The taxonomy of strain Ce-3 based on the Genome Taxonomy Database (release 202) determined by GTBD-Tk v1.7.0 (10) was positioned in the genus *Bradyrhizobium* in the phylum *Proteobacteria*. The strains that were closely related to strain Ce-3 were *Bradyrhizobium* sp. strain C9 (GenBank accession number GCF_002532045.1) and Bradyrhizobium erythrophlei MT12 (GenBank accession number GCF_900105845.1), with average nucleotide identities of 91.86 and 91.79%, respectively.

Information from the genome of strain Ce-3 may help to elucidate new mechanisms for the Ln-dependent phenotypes of the *Bradyrhizobium* genus.

### Data availability.

The draft genome sequence of *Bradyrhizobium* sp. strain Ce-3 was deposited in DDBJ/ENA/GenBank under the accession number BQUV00000000.1. The draft genome project data were submitted under BioProject accession number PRJDB12974, DRA accession number DRA013545, and Sequence Read Archive (SRA) accession number DRX336804.
